# Author Correction: A mixed antagonistic/synergistic miRNA repression model enables accurate predictions of multi-input miRNA sensor activity

**DOI:** 10.1038/s41467-018-07158-1

**Published:** 2018-11-02

**Authors:** Jeremy J. Gam, Jonathan Babb, Ron Weiss

**Affiliations:** 1 Department of Biological Engineering, Massachusetts Institute of Technology (MIT), Cambridge, MA 02139 USA; 2 Synthetic Biology Center, MIT, Cambridge, MA 02139 USA

Correction to: *Nature Communications* 10.1038/s41467-018-04575-0, published online 22 June 2018

In the originally published version of this Article, financial support was not fully acknowledged. The PDF and HTML versions of the Article have now been corrected to include support from the National Science Foundation (NSF), award number 1745645.

