# Supplementary material for: A mixed antagonistic/synergistic miRNA repression model enables accurate predictions of multi-input miRNA sensor activity
Source: Nat Commun. 2018 Jun 22;9:2430. doi: 10.1038/s41467-018-04575-0 (PMC6014984; doi:10.1038/s41467-018-04575-0)
Supplement: Supplementary file 1 — Supplementary Information [file 41467_2018_4575_MOESM1_ESM.pdf]

A mixed antagonistic/synergistic miRNA repression model enables accurate predictions of multi-input miRNA sensor activity

Gam et al.

Supplementary Information

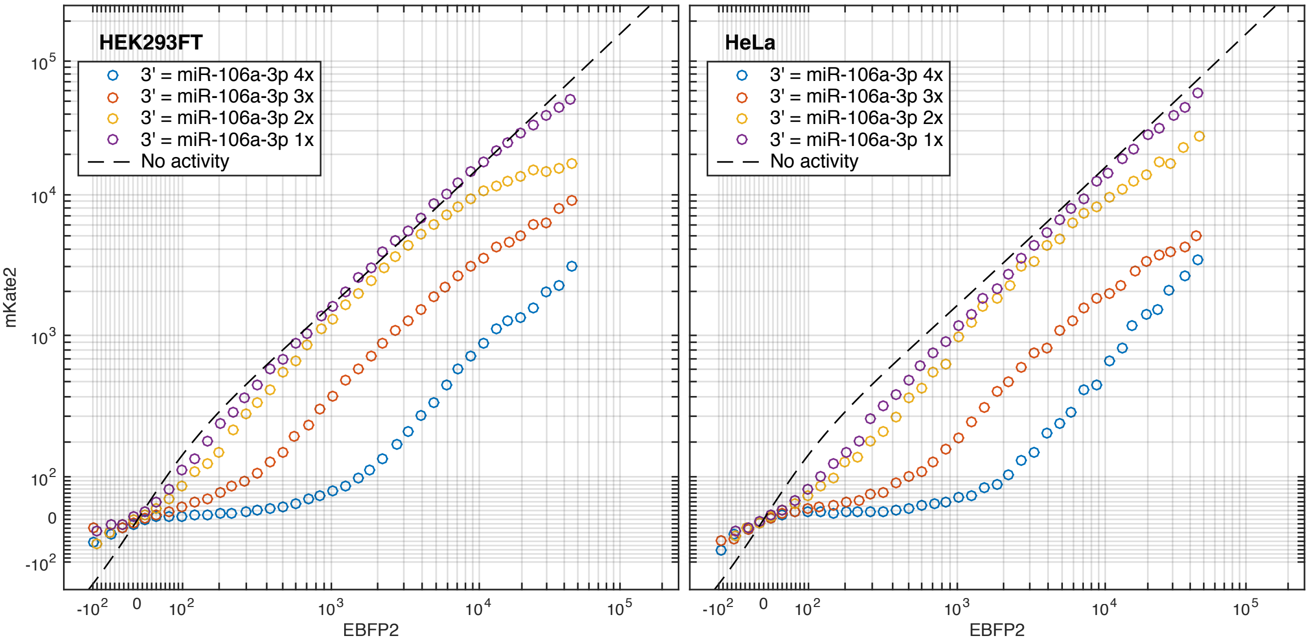

Supplementary Figure 1: Enhanced repression by cooperation with up to four target sites
In both HEK293FT and HeLa cells, combining up to four miR-106a-3p target sites together to form a set results in greater repression. However, further increase from 4x to 8x repeats does not result in further cooperative effects (see figure 2). Interestingly, 1x and 2x repeats show very little activity. These results suggest that the 4x repeats we used for our sensor library is a close to optimal balance that can achieve sufficient repression without incurring excessive DNA synthesis cost.

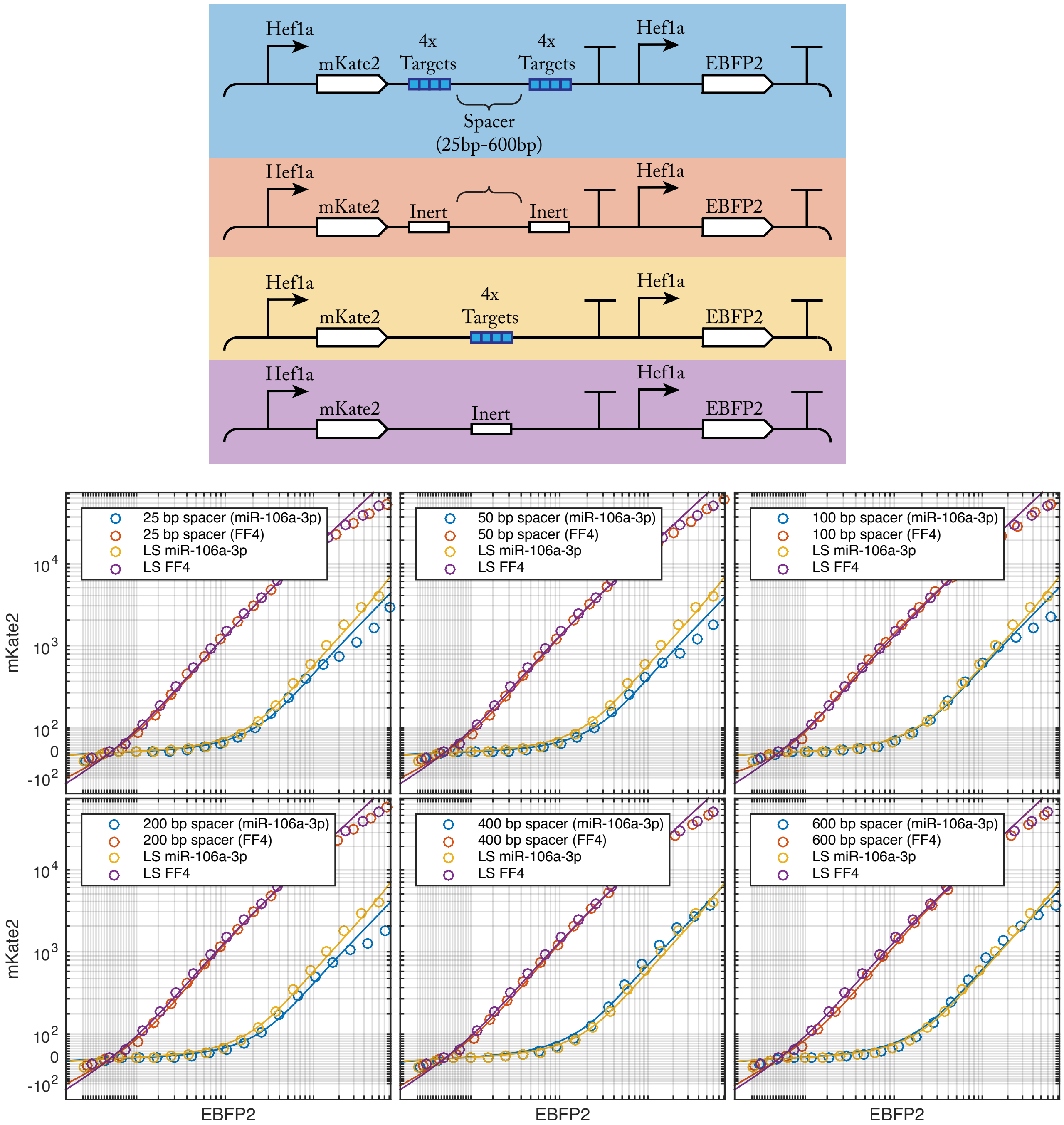

Supplementary Figure 2: Effect of spacer length on miRNA low sensors
To determine whether antagonistic miRNA activities within the 3' UTR are due to steric effects, several variants of a low sensor with miRNA target site sets spaced by different distances were built and tested. For all spacer lengths, sensors with two sets of target sites performed the same as a sensor with only a single set of target sites, indicating almost complete antagonistic miRNA activity for spacers as long as 600 bp - approximately the same length that separates target sites in sensors with target sets across UTRs which show synergistic activity (Fig. 3). This result rules out steric or length-dependent effects for observed antagonism. Assembly was performed using LSBr as backbone and LSB/JG107 providing miRNA target sites, with JG106 providing different length spacers derived from mKate2 coding sequence. Transfections were performed in HEK293FT cells which possess high endogenous miR-106a-3p activity

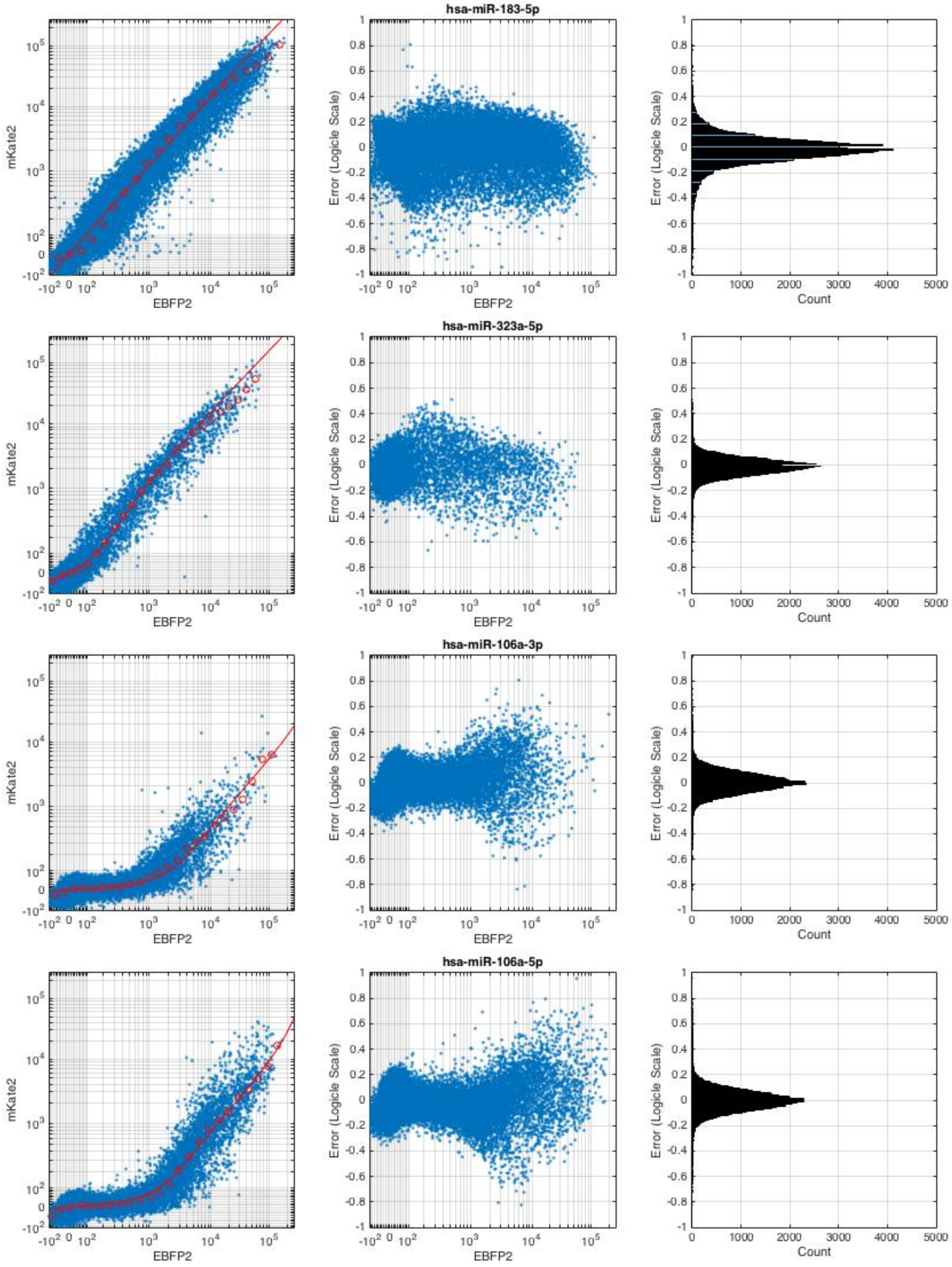

Supplementary Figure 3: Goodness of fit for the miRNA repression model
After transfection of miRNA low sensor libraries into HEK293FT cells, we sought to determine whether observed miRNA activities could be adequately explained by a simple repression model. After fitting M and K_M to the data, residuals and a histogram of the residuals was plotted for several miRNAs with different activities. Residuals are relatively constant across EBFP2 expression levels and centered about zero, with slightly increased variance at high transfection levels. While minor deviations were found in some cases, most of the data is captured by the model.

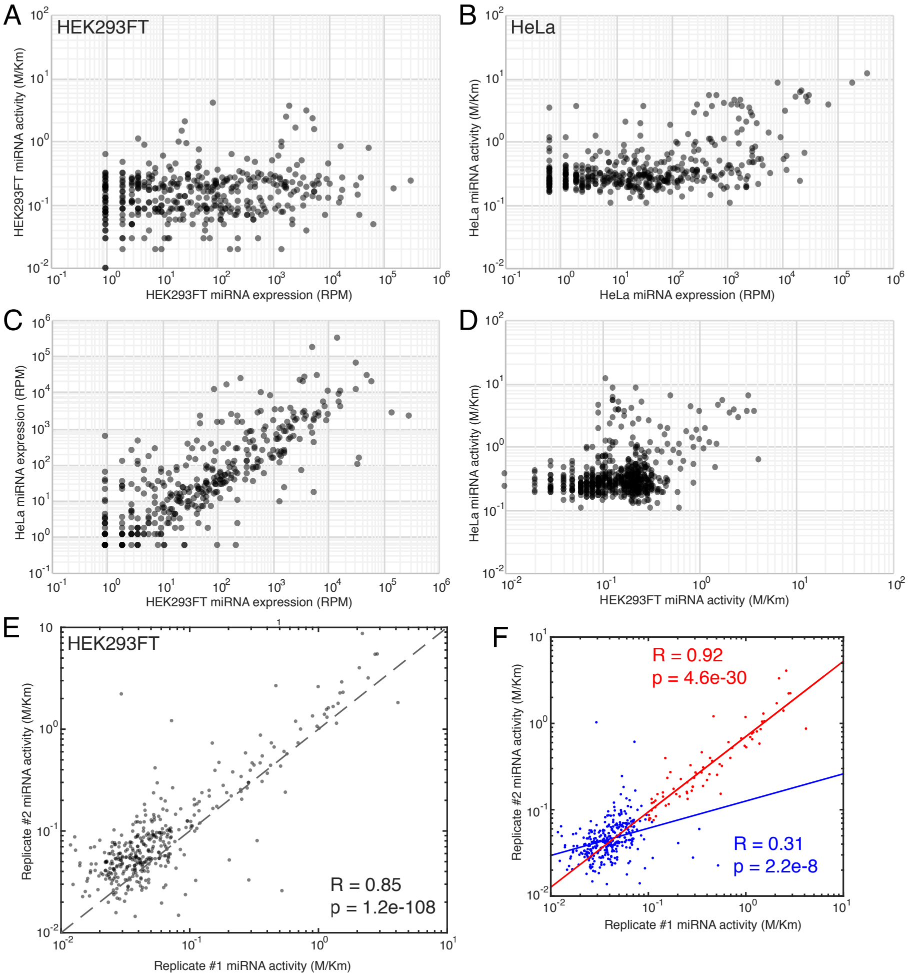

Supplementary Figure 4: Low sensor library data in HEK293FT and HeLa cells
(A,B) A miRNA activity metric of M/K_M derived from parameter fits is plotted against miRNA expression data in reads per million (RPM) obtained by high throughput sequencing. Similarly to other results comparing miRNA activity and abundance, a poor correlation between the two measures is observed for both cell lines tested here. While several hypotheses exist for the discrepancy (ceRNA hypothesis, cellular localization, miRNA modifications) the extent of contributions from each of these and other possible effects is yet to be determined. High throughput sequencing was performed using the NEBNext small RNA kit according to the manufacturer's protocol and sequencing was performed using an Illumina HiSeq 2000. Analysis was performed using cutadapt to trim the 5' SR adapters, fastq-multx to demultiplex, cutadapt to trim the 3' SR adapter and barcodes, followed by miRExpress[6] to quantify miRNA expression based on sequences in miRBase 21.[7] **(C)** miRNA expression for the two cell lines are plotted against each other. Many miRNAs are differentially expressed between the two lines. **(D)** miRNA activity observed using our sensors in HEK293FT and HeLa are plotted. Several miRNAs show greater activity in HeLa vs HEK293FT (HeLa-specific) and a few show greater activity in HEK293FT vs HeLa (HEK293FT-specific). Note the difference between expression data in (C) vs activity data in (D) **(E)** The reproducibility of the M/K_M metric was tested by repeating two biological replicates of reverse transfection in HEK293FT cells and parameter fitting of the low sensor library. The M/K_M metric appears reproducible over the two replicates suggesting M/K_M may be used as a proxy for miRNA activity if a single measure for activity is required. Replicates showed a correlation coefficient of R=0.85 and significantly small p-value, rejecting the null hypothesis that sensor data from replicates was uncorrelated (calculated on log transformed data; linear data also show significant correlation of R=0.82, p=4.5e-95). **(F)** Correlation coefficient and p-values were calculated for groups of either high activity (M/Km >0.1, red) and low activity (M/Km <0.1, blue) sensors in HEK293FT. Both groups show statistically significant correlation though there was a large reduction in correlation for low activity sensors, limiting reproducibility in low activity ranges. However, it should be noted that miRNA activities within this low regime (M/Km between 0.01 and 0.1) behave similarly with little detectable repression. Variance for low activity measurements may be due to noise in model fitting or amplified by the log transform.

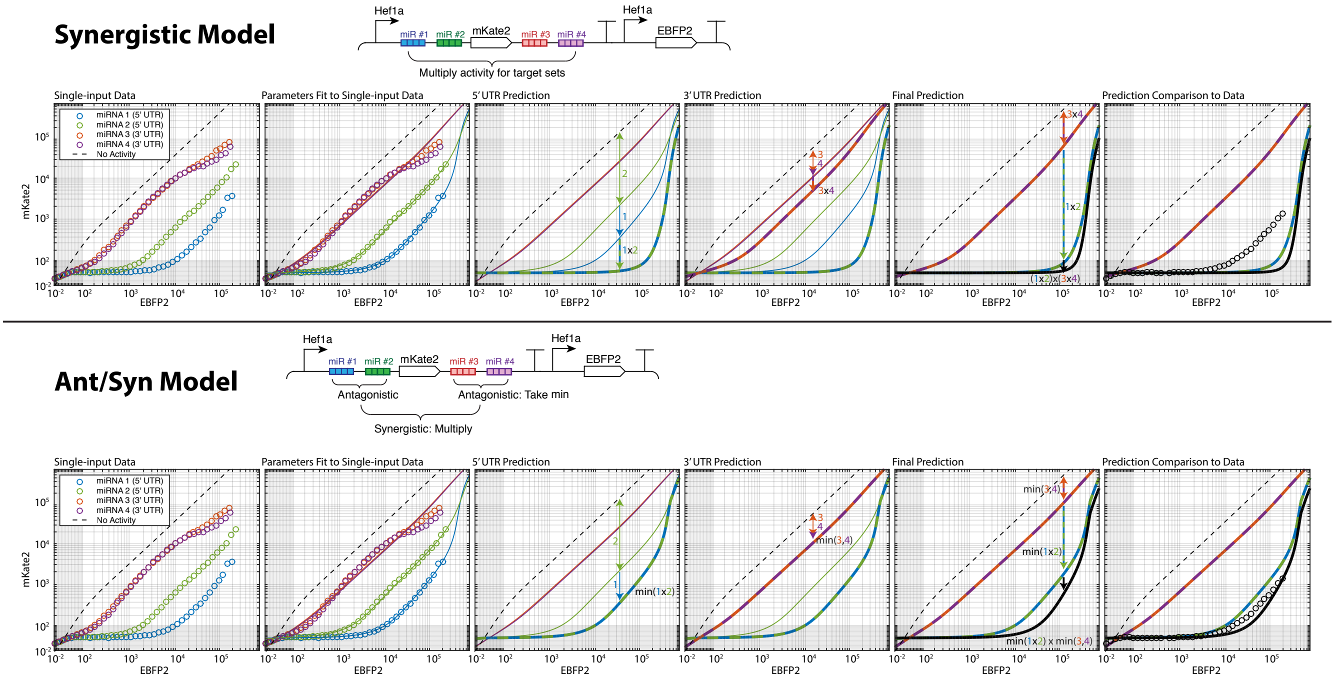

Supplementary Figure 5: Workflow for predictions based on a synergistic-only repression model vs the Ant/Syn model

An illustration of the mathematical operations used to make predictions for synergistic-only vs Ant/Syn models is shown. In this example for HEK293FT cells, miR #1 = hsa-miR-363-3p, miR #2 = hsa-miR-196a-5p, miR #3 = hsa-miR-33b-5p, miR #4 = hsa-miR-340-5p. 1) Single input data are binned according to EBFP2 fluorescence and medians taken. 2) Parameters are fit to the binned data, generating the basis for all predictions using those miRNAs. 3) The 5' UTR prediction is calculated by multiplying the fold changes from miR #1 and miR #2 from the no activity reference point for each EBFP2 fluorescence level (synergistic model) or by taking the minimum mKate2 fluorescence within miR #1 or miR #2 for each EBFP2 level (Ant/Syn model). 4) 3' UTR prediction is made similarly to the 5' UTR prediction except using miR #3 and miR #4. 5) The final prediction is determined by multiplying fold changes from the two separate UTR predictions again relative to the no activity reference. 6) Data observed by transfecting the 4-input sensor construct into cells is compared to the prediction made only from single-input sensor data.

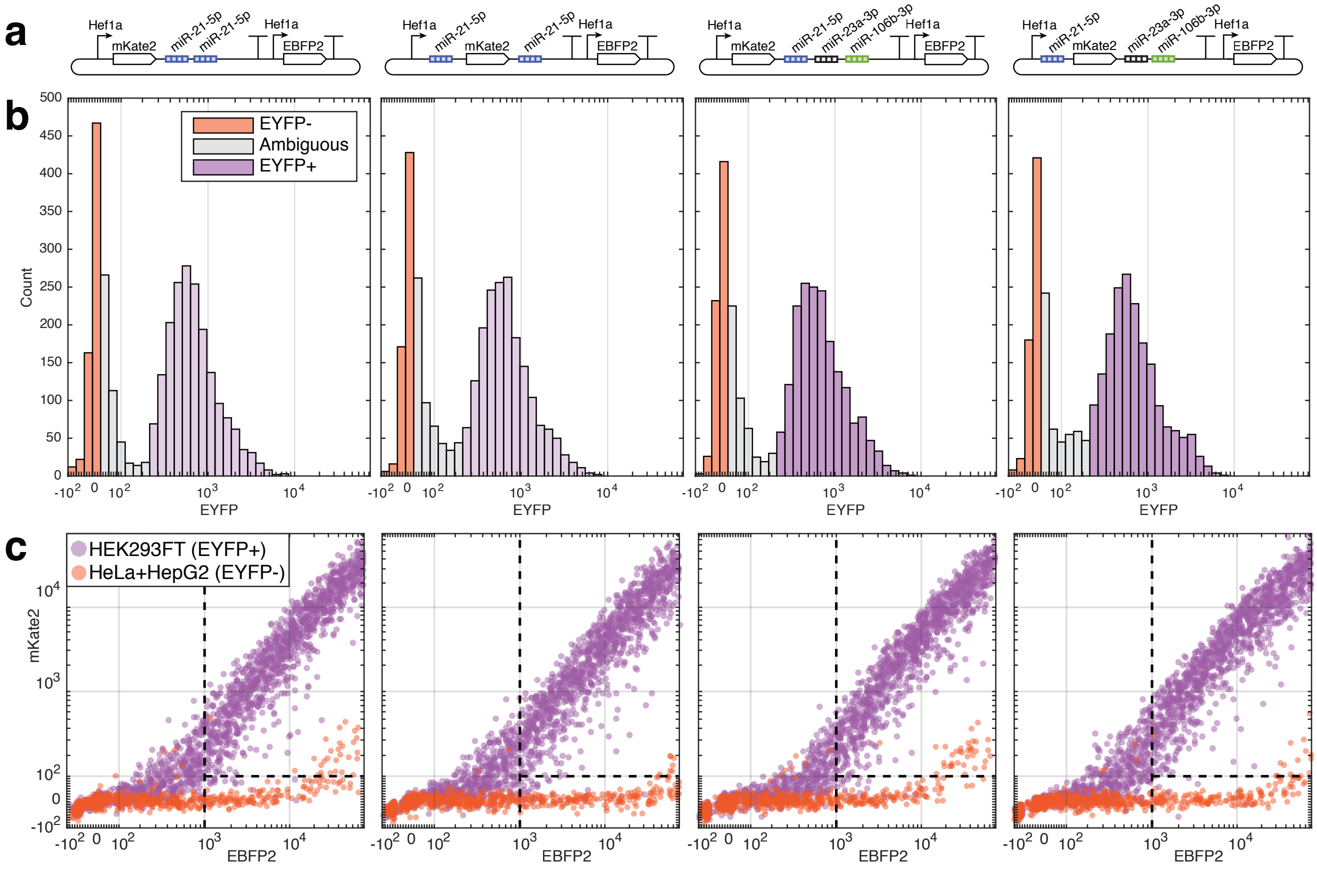

Supplementary Figure 6: Gating of EYFP+ cells (HEK293FT) from EYFP- cells (HeLa and HepG2) We used HEK293FT cells expressing EYFP from the genome to determine whether cells in coculture were indeed HEK293FT or HeLa/HepG2. **(a)** The same sensors and classifiers from Figure 5 are shown. Data for each construct is shown in the column below the respective circuit diagram. **(b)** Histogram of EYFP for cocultured cells is illustrated. Cells that could be unambiguously assigned to EYFP+ and EYFP- populations are colored purple and red respectively. **(c)** Data from Figure 5 with the EYFP+ and EYFP- cells colored in the same fashion as (b).

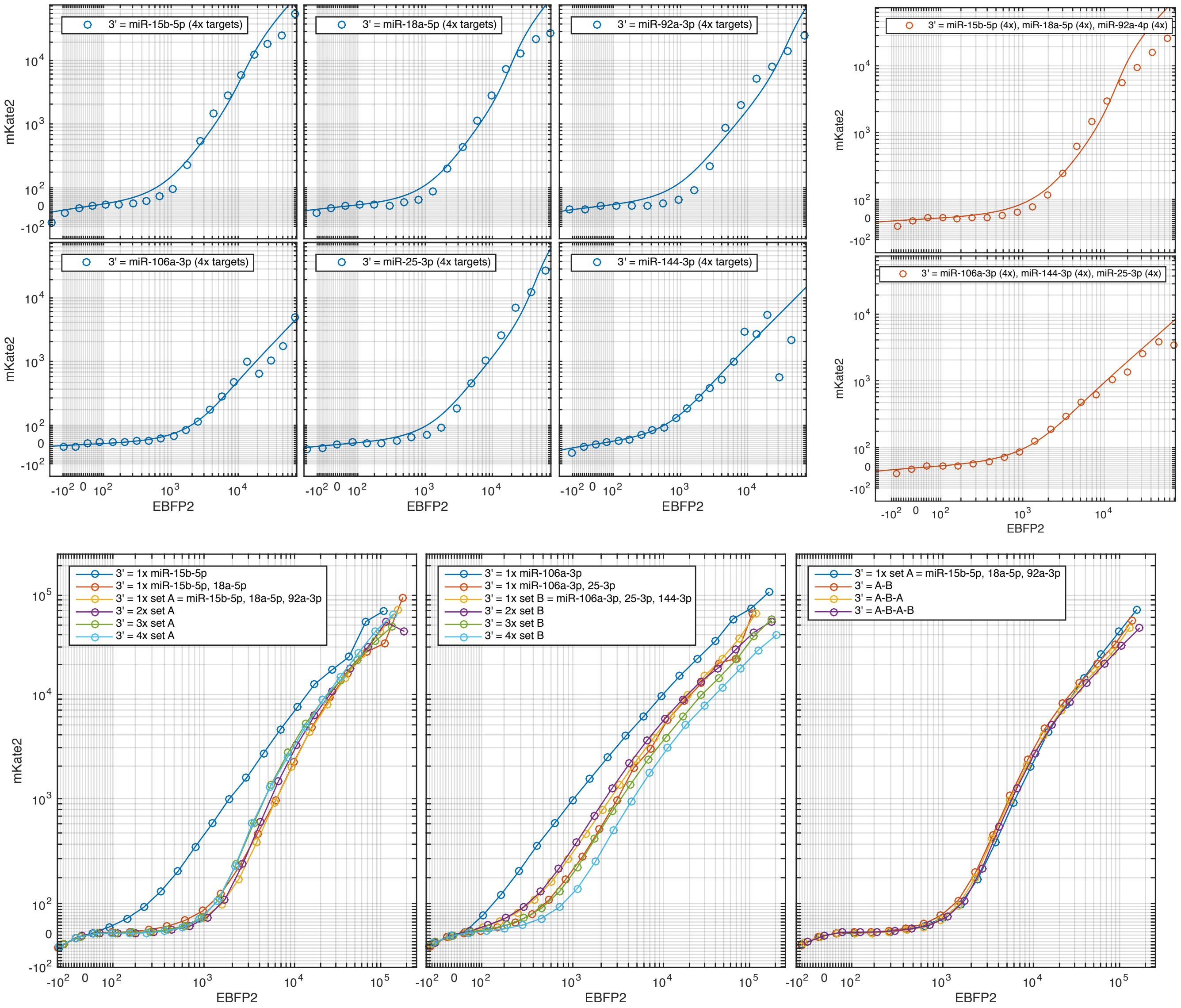

Supplementary Figure 7: Dependent effects are maintained in miRNA sets containing distinct target sites

To further explore the antagonistic activity contributions from sets of miRNA targets, we constructed miRNA sets composed of three different high activity miRNAs. Single input sensor data are plotted (upper left) and data for the corresponding 3-input classifier designs are plotted (upper right) for reference. We observed that concatenating up to four repeats of these target sets had negligible effect on observed activity for set A [miR-15b-5p, miR-18a-5p, miR-92a-3p] and minimal effect for set B [miR-106a-3p, miR-25-3p, miR-144-3p] (bottom). We also tested single repeats for sub sets of miRNAs in sets A and B. We found that at least two target sites were required to see significant repression. A minimal trend towards increased activity with increasing numbers of target sets exists for set B likely due to slightly mismatched activity, as miR-106a-3p has slightly higher activity than either miR-25-3p or miR-144-3p. The maintenance of antagonistic effects even when miRNA targets are different from each other suggests that possible mechanism for antagonism could be strong cooperative effects between Ago molecules that is not dependent on miRNA sequence, which is saturable at high numbers of target sites. One possibility is Ago-TNRC6 complexes containing many Ago molecules allowing for cooperative binding of Agos bearing different miRNA. Further exploration into the extent of complexes containing multiple Agos may be warranted in future studies.

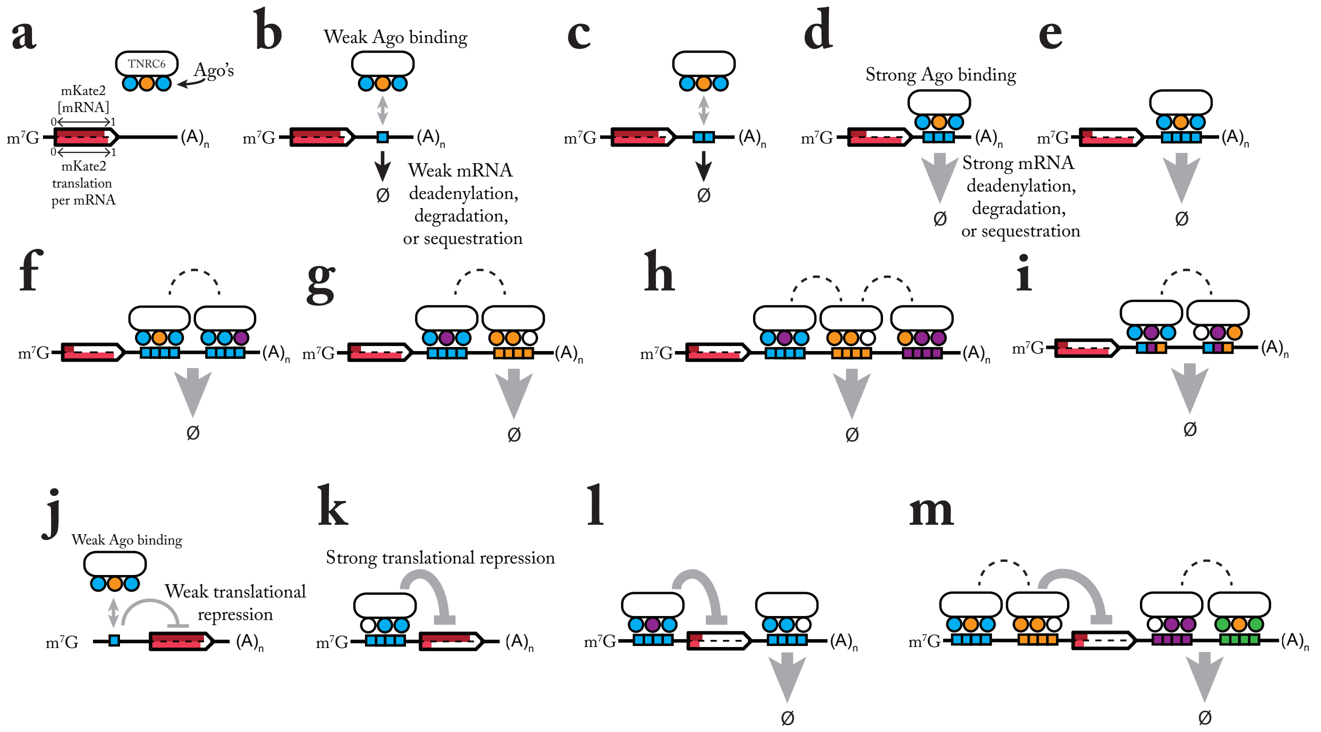

Supplementary Figure 8: Speculative mechanistic model for miRNA repression observed in this study We present a speculative model that explains observations made in this study. Relative mKate reporter mRNA concentrations are shown above the coding sequence cartoon while relative translation levels per mRNA molecule are shown below. Arrow widths denote the amount of flux through a given step and gray arrows show the proposed rate limiting step for a given sensor. Argonaut molecules and miRNA target sites are colored according to miRNA loaded into the Ago molecule or bearing matching sequence to the target respectively. Dotted lines denote Agos that share the same repression machinery. We hypothesize that miRNA target sites within the 3' UTR mainly act to deadenylate, degrade, or sequester mRNA transcripts without affecting translation and vice versa for target sites within the 5' UTR. However the model does not strictly require assignment of these degradation pathways, only that repression mechanisms for the two UTRs be distinct enough that they do not significantly share resources and can separately saturated by addition of many miRNA target sites. Future models may swap or substitute repression mechanisms as further data becomes available. **a-e)** The model involves cooperative binding of Ago to miRNA target sites. This cooperativity is necessary to explain why little repression is observed for sensors bearing 1-2 miRNA target sites but significant repression for those bearing 3-4 or more targets (Supplementary Fig. 1). The data suggest that at low numbers of targets, binding of Ago to reporter transcripts is the rate limiting step - resulting in low numbers of transcripts that can be deadenylated/degraded/sequestered. **f-i)** Addition of up to 8 of the same miRNA target site or 4 each two or three different miRNA target sites in blocks or interleaved fashion all resulted in similar repression to sensors with only 4 strong miRNA targets (Fig. 2, 2, 7). These data suggest that inclusion of target sites above $\sim$4 repeats results in saturation of repression machinery and that degradation is not likely to be simple cleavage mediated by Ago2 as this would be unlikely to result in saturation at such low numbers of target sites. Instead, the data suggest that Ago binds to other factors that repress the transcript and these other factors can be saturated. **j-l)** We observed that miRNA targets within the 5' UTR repress independently (i.e. synergistically) targets within the 3' UTR. We propose that a separate repression mechanism exists for 5' UTR miRNA targets, here we show one possibility: translational repression either at initiation or elongation. With four miRNA targets within the 5' strong repression is observed and with the same set of 4x miRNA targets within the 5' and 3' UTRs a combined effect on repression is observed (Fig. 3). In our speculative model, the combined effect is a result of both reduced mKate2 mRNA concentration and translation levels per mRNA and that binding of Ago to one UTR cooperatively enhances binding to the other UTR. **m)** For our 4-input miRNA sensors, we observed antagonism within the UTRs and synergy across UTRs, again supporting the notion that miRNA target sites within the 5' and 3' UTRs repress by separate mechanisms and are independently saturable (Fig. 4).

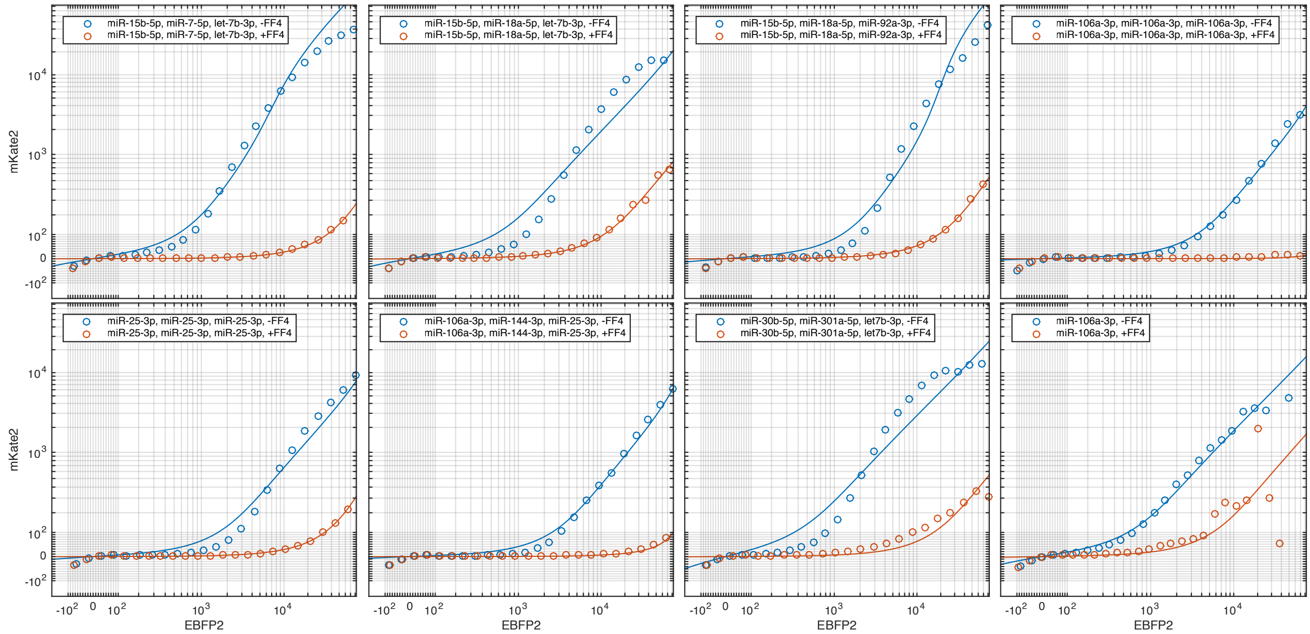

Supplementary Figure 9: Antagonistic interactions not due to repression limits
Several variants of miRNA sensors bearing sets of 4x miRNA target repeats and also a set of 4x FF4 target sites within the 3' UTR were tested in HEK293FT cells with and without FF4 siRNA. In all cases, addition of FF4 siRNA was able to further knockdown mKate2 expression, showing that repression is not complete with three sets of high activity miRNA targets. Interestingly, FF4 knockdown seemed to take place in addition to knockdown from endogenous miRNAs (i.e. more synergistic rather than antagonistic), suggesting distinct mechanisms for reporter repression with siRNA and miRNA.

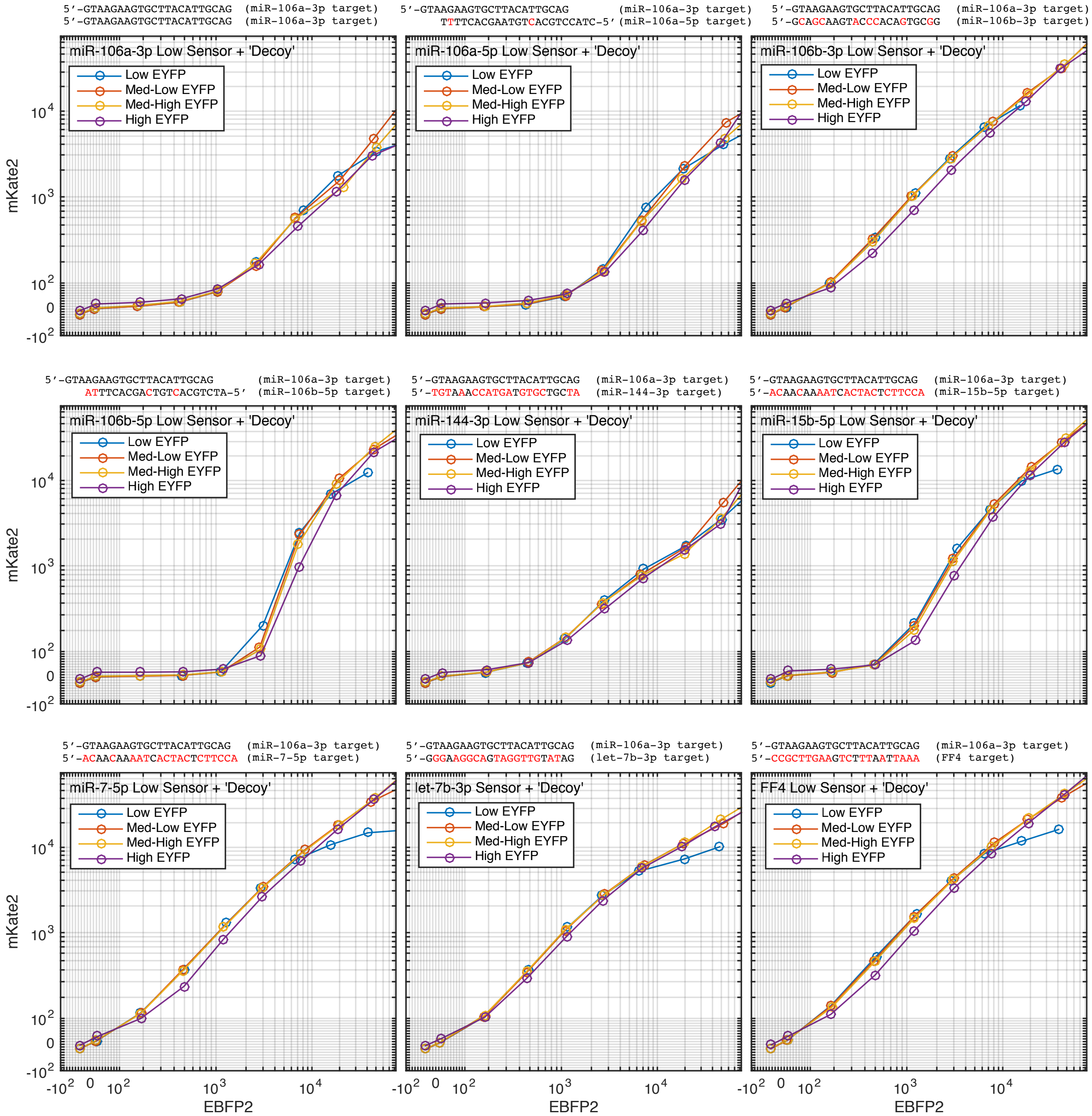

Supplementary Figure 10: Minimal resource sharing between miRNA sensors of related and unrelated miRNAs

To test whether transfection of miRNA sensors has measurable effect on repression of other miRNA targets, a 'decoy' sensor encoding miR-106a-3p target sites along with a separate hEF1a-EYFP transfection marker was built. Sequential transfection of the decoy sensor followed by one of nine low sensors was conducted in HEK293FT cells. Cells were binned from low to high EYFP expression - indicating low to high levels of decoy sensor - and traditional EBFP2 vs mKate2 plots were constructed. For all tested sensors, including related (miR-106a-3p, miR-106a-5p, miR-106b-3p, miR-106b-5p) and unrelated (miR-144-3p, miR-15b-5p, miR-7-5p, let-7b-3p) sensors, no trend was seen across different decoy sensor levels. If resource sharing was apparent, less miRNA activity would be observed for higher levels of decoy sensor, but this was not the case. These results suggest that miRNA sensors impart low or undetectable levels of resource sharing on other targets.

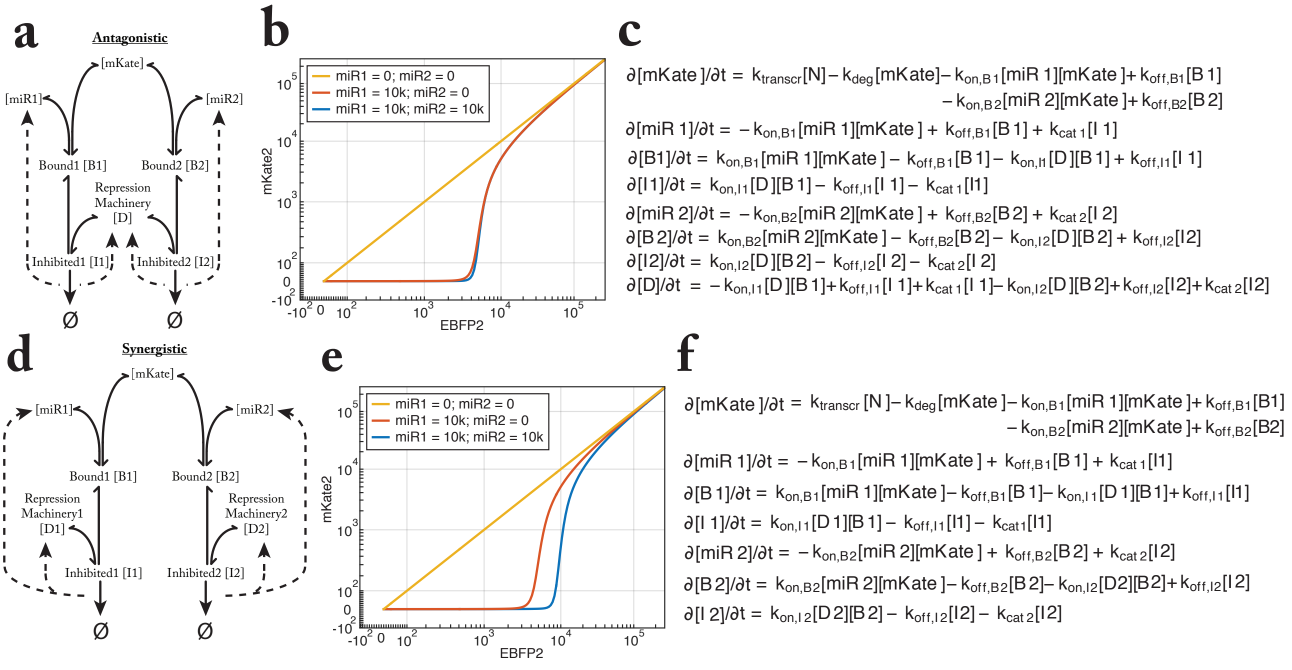

Supplementary Figure 11: Speculative ODE model for antagonistic and synergistic repression We propose general ordinary differential equation (ODE) models describing repression from miRNAs when repression machinery is shared, generating an antagonistic interaction, or distinct, generating a synergistic interaction. **a)** The model for antagonistic interactions includes mKate transcripts that can be reversibly bound by different miRNA-RISC molecules [miR1] and [miR2] to form bound complexes. The bound complexes can then form inhibited complexes after reversible binding with a shared pool of repression machinery. Inhibited complexes can also be irreversibly degraded, eliminating the mKate transcript and regenerating the miRNA and repression machinery. **b)** Results from a numerical simulation of the model described in (a) with two similar miRNAs. miRNAs are present at concentrations of either 0 or 10,000 molecules per cell. As expected from an antagonistic interaction, the addition of miR2 results in minimal further repression compared to miR1 alone since the repression machinery is saturated at concentrations provided by miR1 itself. **c)** The ODEs used in (a) and (b) are listed here for reference. **d)** The model for synergistic interactions is similar to that for the antagonistic model, with the exception that the repression machinery for miR1 and miR2 are distinct, since in this case miR1 and miR2 would have target sites in opposite UTRs (in this example miR1 in the 5' UTR and miR2 in the 3' UTR). Note that initial $D2 = D$ for the antagonistic model, since both D2 and D2 are concentrations for the repression machinery associated with the 3' UTR. For simplicity, we also set $D1 = D$ since we generally observe that repression from 5' target sites is similar to that from 3' target sites, though this equivalence is not required for a synergistic interaction to be observed. **e)** In the simulation corresponding to the model in (d), miR2 is able to contribute to further repression of mKate compared to miR1 alone, since repression machinery not shared between the two miRNAs. This behavior is descriptive of a synergistic relationship. **f)** The ODEs used in (d) and (e) are listed here. Equations are similar to (c) except that there are two terms for repression machinery [D1] and [D2].

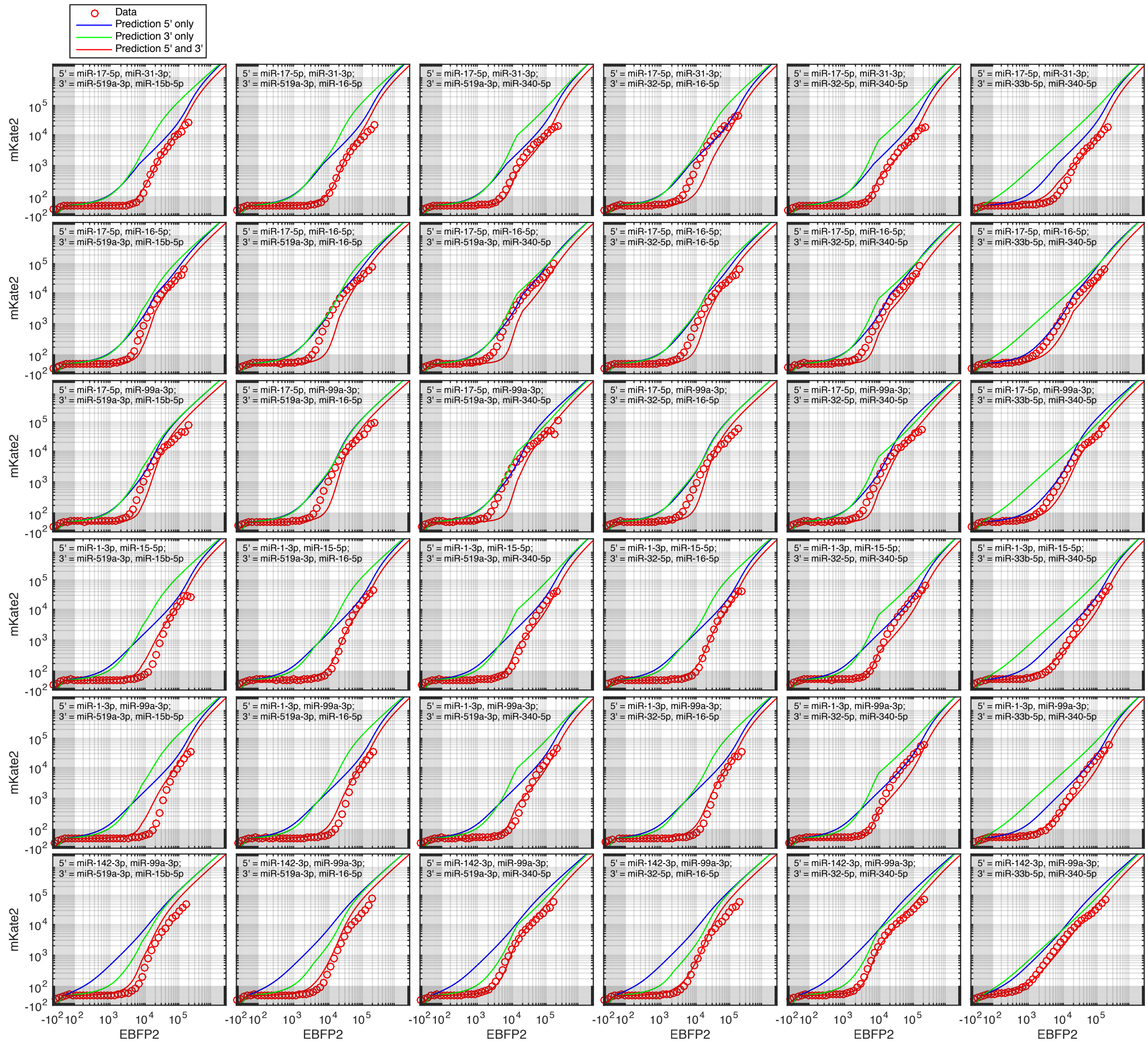

Supplementary Figure 12: Predictions for Ant/Syn model

miRNA activity data and predictions using Ant/Syn model for 36 different 4-input classifiers. Predictions are shown for 5' UTR only (blue lines), 3' UTR only (green lines), combined prediction (red lines), and data (red circles). In general, predictions explain observed miRNA activity well.

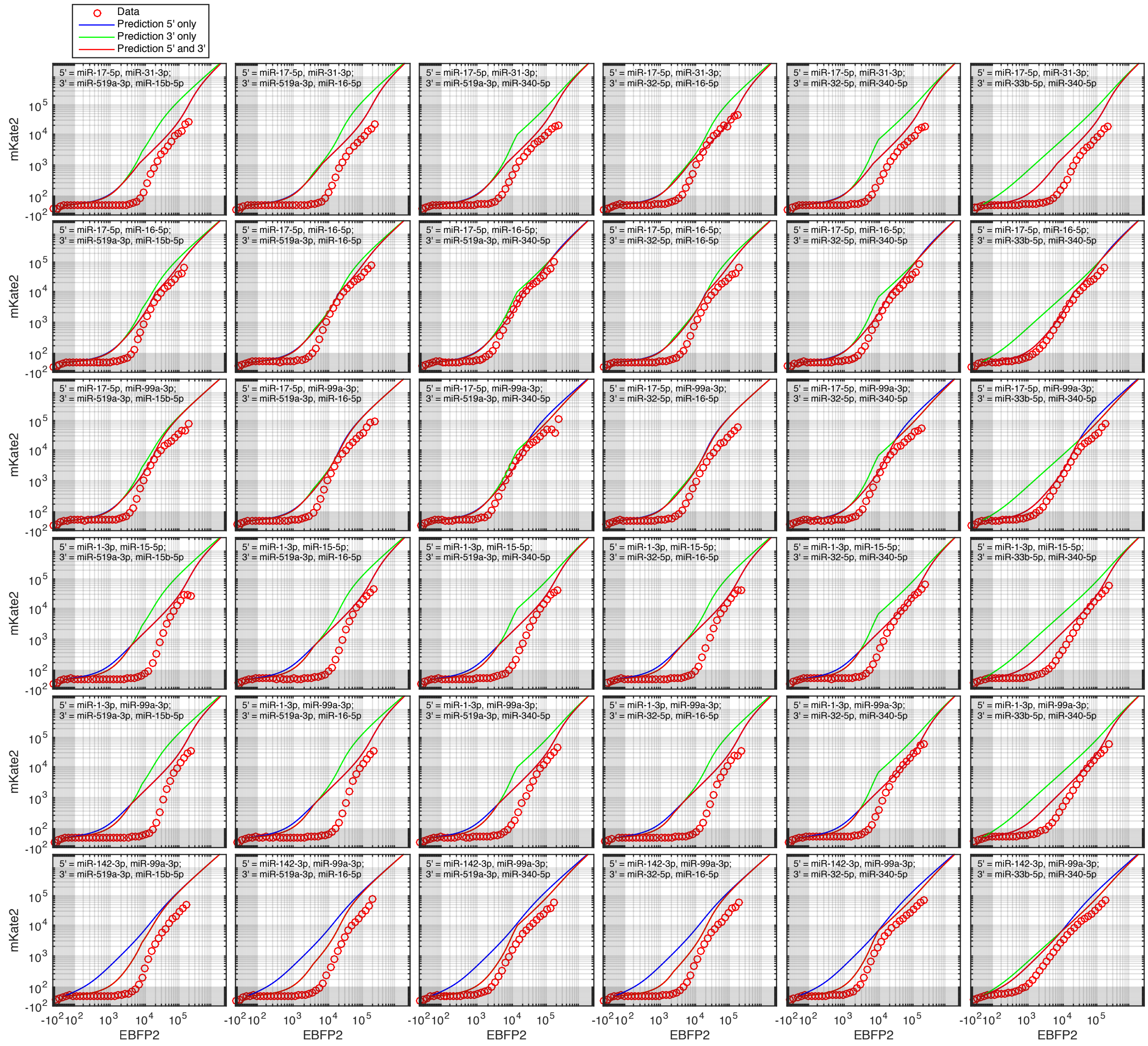

Supplementary Figure 13: Predictions for antagonist-only model
miRNA activity data and predictions using antagonistic-only model for 36 different 4-input classifiers. Predictions are shown for 5' UTR only (blue lines), 3' UTR only (green lines), combined prediction (red lines), and data (red circles). In general, predictions generated by the antagonistic-only model underestimate observed miRNA activity.

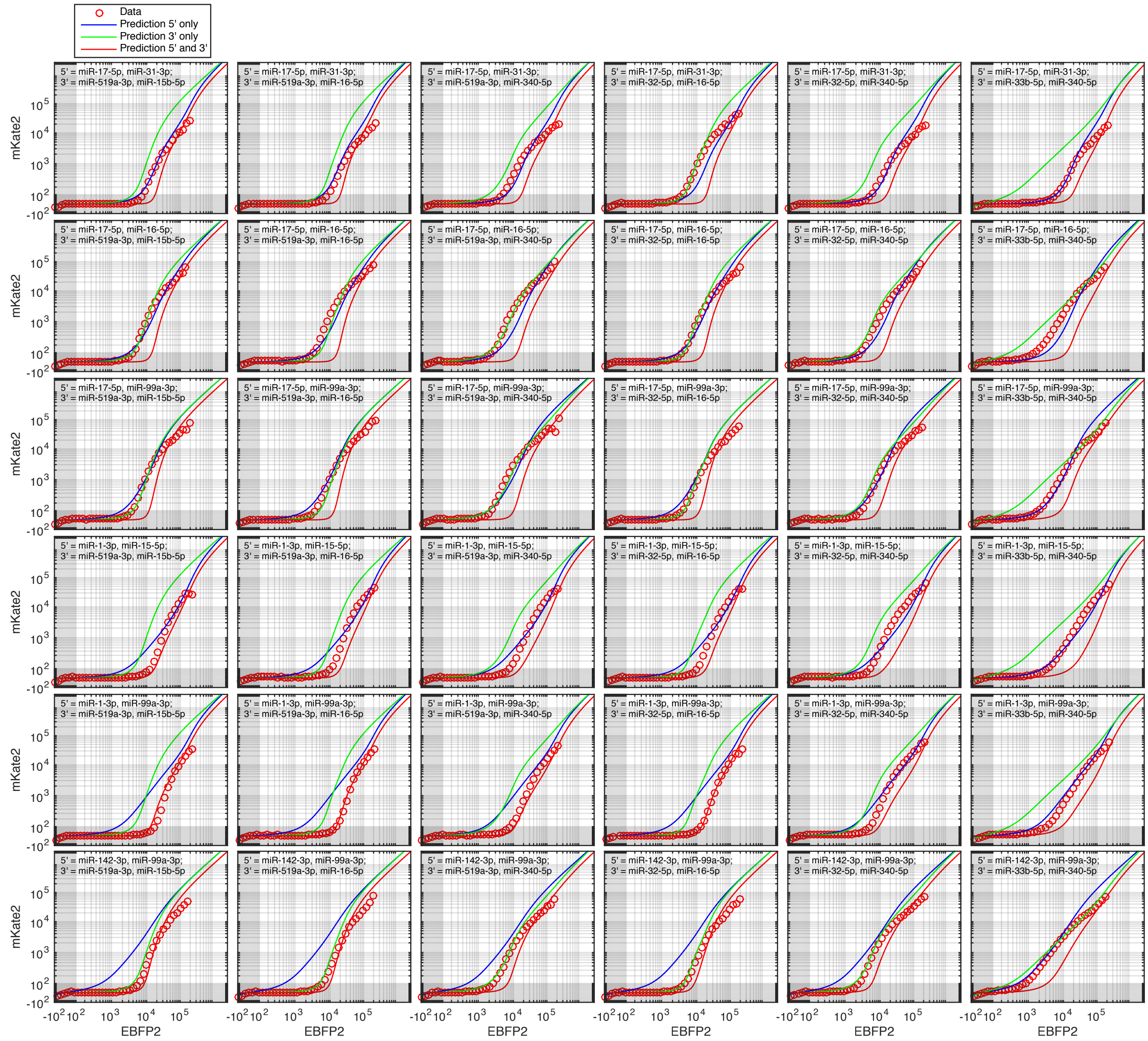

Supplementary Figure 14: Predictions for the synergistic-only model
miRNA activity data and predictions using synergistic-only model for 36 different 4-input classifiers. Predictions are shown for 5' UTR only (blue lines), 3' UTR only (green lines), combined prediction (red lines), and data (red circles). In general, predictions generated by the synergistic-only model overestimate observed miRNA activity.

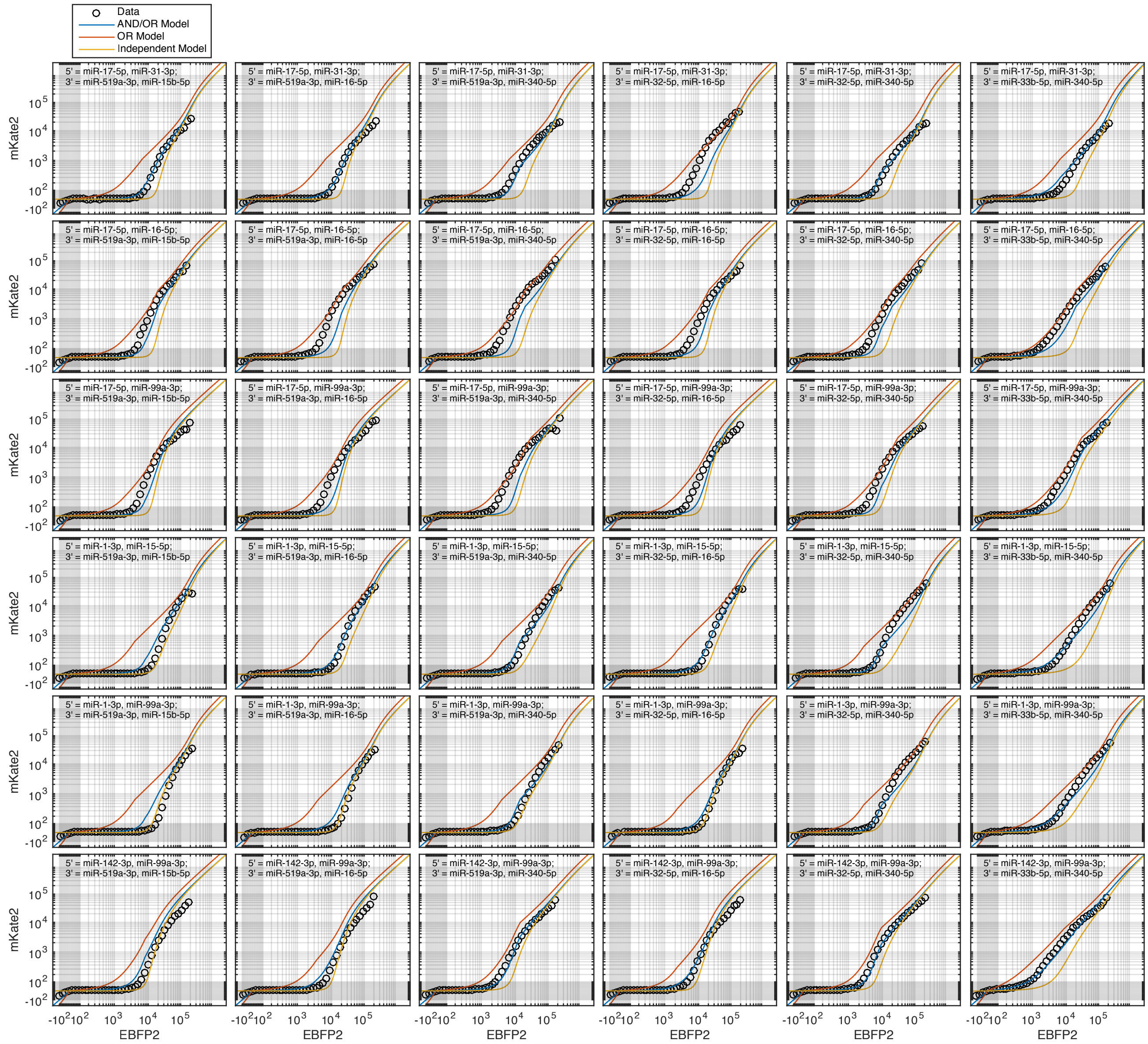

Supplementary Figure 15: Comparison of predictions for all three models
Direct comparison between the three models for 36 different 4-input classifiers. In most cases, the Ant/Syn model best explains the data while the antagonistic-only model underestimates activity and the synergistic-only model overestimates activity.

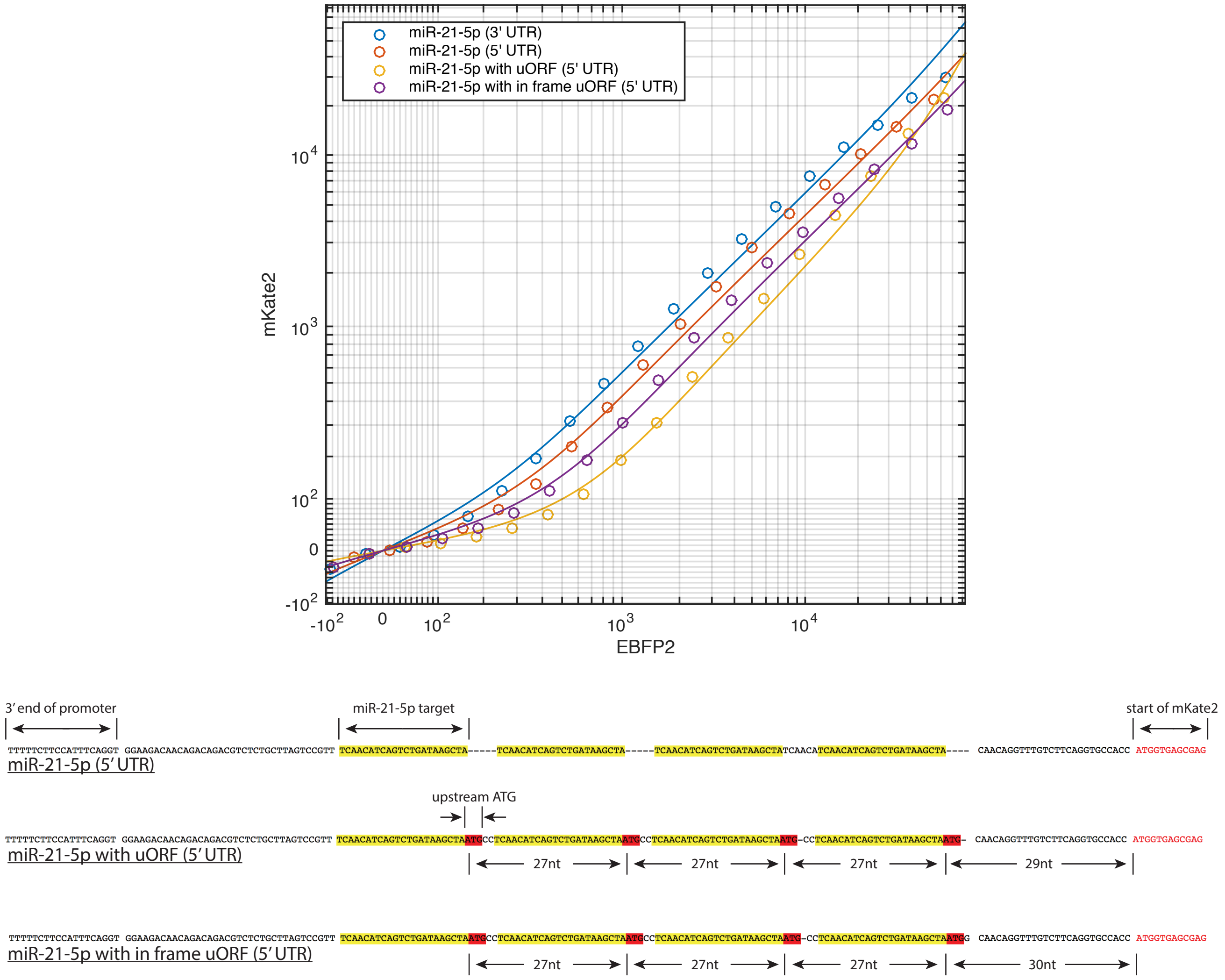

Supplementary Figure 16: Repression of sensors bearing targets with and without uORFs
miRNA low sensors were constructed bearing four repeats of miR-21-5p in the 3' UTR (blue circles), or 5' UTR (red, yellow, purple circles). Since miR-21-5p does not have ATGs, several ATGs were added between the miR-21-5p target sites in some sensors. Extra bases were added to separate the ATGs by a number of bases divisible by three. The distance between the last ATG and the true reporter start codon was either divisible by three (purple circles) or not (yellow circles). We observed a 2-fold drop in fluorescence when out of frame ATGs were added that was partially recovered when the ATGs were then shifted into frame. When using miRNA targets containing ATGs, altering the sensor design to keep them in frame may allow better measurements of miRNA activity.

**HEK293FT**	**HeLa**	**HepG2**	**miRNAs**
High	Low	Low	miR-16-5p, miR-18a-5p, miR-519c-3p, miR-520c-3p
Low	High	Low	miR-27b-3p, miR-29b-3p, miR-98-5p
Low	Low	High	miR-16-5p, miR-10a-3p, miR-106b-3p, miR-142-5p
Low	High	High	miR-16-5p, miR-21, miR-23a-3p, miR-130a-3p, miR-29c-3p

Supplementary Table 1: Best candidate miRNAs with specific activities among tested cell lines. miRNAs listed here were considered for construction in HEK293FT classifiers. Classifiers with highest on/off predicted by the Ant/Syn model were constructed and tested in cells.

**Plasmid Name**<br>UTR Location<br>GG Overhang	**miRNA 1**<br>5' UTR<br>Q3-Q5	**miRNA 2**<br>5' UTR<br>Q5-Q9	**miRNA 3**<br>3' UTR<br>Q1-Q3	**miRNA 4**<br>3' UTR<br>Q3-Q2
JG324	miR-17-5p (H)	miR-31-3p (H)	miR-519a-3p (H)	miR-15b-5p (H)
JG325	miR-17-5p (H)	miR-31-3p (H)	miR-519a-3p (H)	miR-16-5p (M)
JG326	miR-17-5p (H)	miR-31-3p (H)	miR-519a-3p (H)	miR-340-5p (L)
JG327	miR-17-5p (H)	miR-31-3p (H)	miR-32-5p (M)	miR-16-5p (M)
JG328	miR-17-5p (H)	miR-31-3p (H)	miR-32-5p (M)	miR-340-5p (L)
JG329	miR-17-5p (H)	miR-31-3p (H)	miR-33b-5p (L)	miR-340-5p (L)
JG330	miR-17-5p (H)	miR-16-5p (M)	miR-519a-3p (H)	miR-15b-5p (H)
JG331	miR-17-5p (H)	miR-16-5p (M)	miR-519a-3p (H)	miR-16-5p (M)
JG332	miR-17-5p (H)	miR-16-5p (M)	miR-519a-3p (H)	miR-340-5p (L)
JG333	miR-17-5p (H)	miR-16-5p (M)	miR-32-5p (M)	miR-16-5p (M)
JG334	miR-17-5p (H)	miR-16-5p (M)	miR-32-5p (M)	miR-340-5p (L)
JG335	miR-17-5p (H)	miR-16-5p (M)	miR-33b-5p (L)	miR-340-5p (L)
JG336	miR-17-5p (H)	miR-99a-3p (L)	miR-519a-3p (H)	miR-15b-5p (H)
JG337	miR-17-5p (H)	miR-99a-3p (L)	miR-519a-3p (H)	miR-16-5p (M)
JG338	miR-17-5p (H)	miR-99a-3p (L)	miR-519a-3p (H)	miR-340-5p (L)
JG339	miR-17-5p (H)	miR-99a-3p (L)	miR-32-5p (M)	miR-16-5p (M)
JG340	miR-17-5p (H)	miR-99a-3p (L)	miR-32-5p (M)	miR-340-5p (L)
JG341	miR-17-5p (H)	miR-99a-3p (L)	miR-33b-5p (L)	miR-340-5p (L)
JG342	miR-1-3p (M)	miR-16-5p (M)	miR-519a-3p (H)	miR-15b-5p (H)
JG343	miR-1-3p (M)	miR-16-5p (M)	miR-519a-3p (H)	miR-16-5p (M)
JG344	miR-1-3p (M)	miR-16-5p (M)	miR-519a-3p (H)	miR-340-5p (L)
JG345	miR-1-3p (M)	miR-16-5p (M)	miR-32-5p (M)	miR-16-5p (M)
JG346	miR-1-3p (M)	miR-16-5p (M)	miR-32-5p (M)	miR-340-5p (L)
JG347	miR-1-3p (M)	miR-16-5p (M)	miR-33b-5p (L)	miR-340-5p (L)
JG348	miR-1-3p (M)	miR-99a-3p (L)	miR-519a-3p (H)	miR-15b-5p (H)
JG349	miR-1-3p (M)	miR-99a-3p (L)	miR-519a-3p (H)	miR-16-5p (M)
JG350	miR-1-3p (M)	miR-99a-3p (L)	miR-519a-3p (H)	miR-340-5p (L)
JG351	miR-1-3p (M)	miR-99a-3p (L)	miR-32-5p (M)	miR-16-5p (M)
JG352	miR-1-3p (M)	miR-99a-3p (L)	miR-32-5p (M)	miR-340-5p (L)
JG353	miR-1-3p (M)	miR-99a-3p (L)	miR-33b-5p (L)	miR-340-5p (L)
JG354	miR-142-3p (L)	miR-99a-3p (L)	miR-519a-3p (H)	miR-15b-5p (H)
JG355	miR-142-3p (L)	miR-99a-3p (L)	miR-519a-3p (H)	miR-16-5p (M)
JG356	miR-142-3p (L)	miR-99a-3p (L)	miR-519a-3p (H)	miR-340-5p (L)
JG357	miR-142-3p (L)	miR-99a-3p (L)	miR-32-5p (M)	miR-16-5p (M)
JG358	miR-142-3p (L)	miR-99a-3p (L)	miR-32-5p (M)	miR-340-5p (L)
JG359	miR-142-3p (L)	miR-99a-3p (L)	miR-33b-5p (L)	miR-340-5p (L)

Supplementary Table 2: miRNA target combinations for sensors with targets in the 5' UTR. miRNA names, UTR location, and golden gate overhangs are listed. miRNA activities in HEK293FT cells are indicated as high (H), medium (M), or low (L)

Rate	Value	Unit (per cell)	Description	Reference
k_{trs}	7	mRNAs/hour	transcription rate	[8,9]
$k_{deg,m}$	0.5	1/hour	mRNA degradation rate	[10,11]
$k_{tln,EBFP2}$	5	proteins/mRNA/hour	EBFP2 translation rate	[9]
$k_{tln,mKate2}$	5	proteins/mRNA/hour	mKate2 translation rate	[9]
$k_{deg,EBFP2}$	0.5	1/hour	EBFP2 degradation rate	[12]
$k_{deg,mKate2}$	0.5	1/hour	mKate2 degradation rate	[12]
K_M	various	molecules	Michaelis constant	[13]
M	various	molecules	total effective miRNA concentration	[14]

Supplementary Table 3: Rate constants used for miRNA repression model

Designation	Sequence	Description
Q1	GCTT	miRNA target overhang 1
Q2	CAAC	miRNA target overhang 2
Q3	CAGA	miRNA target overhang 3
Q4	TGTG	miRNA target overhang 4
Q5	GAGC	miRNA target overhang 5
Q6	AACG	miRNA target overhang 6
Q7	CTTC	miRNA target overhang 7
Q8	AGAC	miRNA target overhang 8
Q9	AGGT	miRNA target overhang 9
Qa	GGAG	overhang between backbone and insulator
Qb	TACT	overhang between insulator and promoter
Qc	CAGA	overhang between promoter and 5' UTR
Qd	AGGT	overhang between 5' UTR and gene
Qe	GCTT	overhang between gene and 3' UTR
Qf	CAAC	overhang between 3' UTR and poly A
Qg	CGCT	overhang between poly A and backbone

Supplementary Table 4: Golden Gate overhangs used for assembly

Supplementary Discussion

miRNA repression mechanisms are varied and complex, with molecular players including the Argonaut proteins, GW182, PABPC, the CCR-NOT complex, the PAN2-PAN3 complex, and decapping proteins.[1] Recent evidence shows even more possible interactions with DDX6, phosphorylation by CSNK1A1 and dephosphorylation by the ANKRD52-PPP6C complex.[2,3] Further complicating matters is the fact that miRNAs can mediate multiple modes of regulation including deadenylation, decapping, cleavage, and translational repression. Our results previous preliminary findings that the repression mechanisms for targets in the 5' UTR and 3' UTR may be distinct (e.g. translational repression in the 5' UTR and mRNA destabilization in the 3' UTR).[4]

Also, we hypothesize that antagonistic interactions could act to reduce noise and increase redundancy in highly regulated genes, since if the miRNAs have similar activity, a decrease in activity of any single miRNA would not have significant impact on gene expression. This is in contrast to the stereotypical additive or independent (i.e. synergistic) models where fluctuation of any single miRNA would have effects on gene expression, propagation of noisy miRNA activity toward target gene expression. This mechanism of noise reduction likely works in tandem with other modes where miRNAs can suppress noise at the network level.[5] As such, it may be possible that highly miRNA-regulated genes use antagonistic interactions within the same UTR to help reject noise and use synergistic interactions across UTRs to increase repression. An ideal future model would be able to take a given UTR sequence (either endogenous or a synthetic design) and predict the extent of repression expected from miRNA regulation. Such a model would need to be able to find miRNA target sites computationally, determine the degree of binding and repression mode based on sequence complementarity, and also predict synergistic, additive, or antagonistic effects from multiple target site interactions. Other requirements would be measurements of miRNA concentration and activity in cell lines of interest. Our work here serves as a step forward for understanding target site interactions in the simplest and highest repressing synthetic systems, in addition to contributing data about miRNA concentration and activity. While our work was oriented towards sensing miRNAs for use in applications like nucleic acid-based therapeutics, we were able to explore some aspects of miRNA biology in the process. For we were able to validate that miRNA targets in the 5' UTR can contribute synergistically to canonical targets in the 3' UTR. We anticipate that future work can build upon the Ant/Syn model to include more diverse types of sequences including those found endogenously (e.g. different target site architectures, UTRs that contain between 1-4 target sites), in order to eventually be able to predict miRNA repression of any UTR.

Supplementary Note 1

The model as described includes reactions listed in equations 1 to 5. In each cell, the number of plasmids is assumed to be a constant value such that the steady state approximation can be made.

$$k_{trs}N - k_{deg,m}[m_{EBFP2}] = 0 \tag{1}$$

$$k_{tln,EBFP2}[m_{EBFP2}] - k_{deg,EBFP2}[EBFP2] = 0 \tag{2}$$

$$k_{trs}N - k_{deg,m}[m_{mKate2}] - k_{on,miR}[m_{mKate2}][m_{miR}] + k_{off}[m_{mKate2,miR}] = 0 \tag{3}$$

$$k_{tln,mKate2}[m_{mKate2}] - k_{deg,mKate2}[mKate2] = 0 \tag{4}$$

$$k_{on,miR}[m_{mKate2}][m_{miR}] - k_{off}[m_{mKate2,miR}] - k_{cat}[m_{mKate2,miR}] = 0 \tag{5}$$

The total concentration of miRNA is the summation of both free (m_{miR}) and bound ($m_{mKate2,miR}$) species. The Michaelis constant is used as a lump parameter, fitting only two parameters to characterize miRNA activity minimizes the risk of overfitting.

$$M = [m_{miR}] + [m_{mKate2,miR}] \tag{6}$$

$$K_m = \frac{k_{cat} + k_{off}}{k_{on}} \tag{7}$$

Use of additional lumped parameters α to δ simplifies the form of equation 12. These lumped parameters have constant value for all miRNA sensors regardless of miRNA activity since they depend only on rates of transcription, translation, and degradation of unbound mKate2 and EBFP2 species.

$$\alpha = \frac{k_{trs}}{k_{deg,m}} \tag{8}$$

$$\beta = \frac{k_{cat}}{k_{deg,m}} \tag{9}$$

$$N = \frac{k_{deg,m} k_{deg,EBFP2}}{k_{trs} k_{tln,EBFP2}} [EBFP2] = \gamma [EBFP2] \tag{10}$$

$$[mKate2] = \frac{k_{tln,EBFP2}}{k_{deg,mKate2}} [m_{mKate2}] = \delta [EBFP2] \tag{11}$$

The solution for mKate2 concentration takes a quadratic form for a single-input miRNA sensor. The two parameters quantifying miRNA activity are M and K_m.

$$[mKate2] = \frac{\delta}{2} (\sqrt{(-\alpha\gamma[EBFP2] + \beta * M + K_m)^2 + 4\alpha\gamma K_m [EBFP2]} + \alpha\gamma[EBFP2] - \beta M - K_m) \tag{12}$$

To obtain predictions for combined miRNA activity for two-input (or more) sensors, several approaches can be taken. While numerical simulation could be used, its implementation would be relatively slow here. We chose to use a more general approach borrowed from combination of (drug) inhibitors where combinations are divided into three classes of interactions: additive - where effects from each inhibitor alone can be 'summed' to obtain the combined effect since inhibitors are assumed not to interact with each other, synergy - where the combined effect is greater than the sum of each inhibitor alone, and antagonism - where the combined effect is less than the sum of each inhibitor alone. General equations for combining miRNA activities using these three classes of interactions have been previously described[15] and are given as follows, where $m_{add.}$, $m_{synerg.}$, and $m_{antag.}$ refer to mKate2 concentrations resulting from the combination of i different miRNAs which individually repress mKate2 concentration to levels m_i. $m_{neg.}$ refers to the mKate2 concentration in the negative control case where the sensor contains no miRNA target sites. For the antagonistic case, the limit as the Hill coefficient (n) approaches zero is taken since that is the case of perfect antagonism, where combined activity simply reflects the highest activity present in the individual sensors. Thus equation 15 is derived from the more general equation 17 for multiple inhibition in Chou-Talalay.[30] Values for $m_{add.}$, $m_{synerg.}$, and $m_{antag.}$ are predicted across the entire range of EBFP2 expression (which serves as a transfection marker) since repression of mKate2 is often threshold-like and dependent on transfection levels. Example predictions using the three equations below for 2-miRNA cases are shown in Figure 2. Also shown are simplified equations for the 2-miRNA case to provide a more intuitive definition of antagonistic, additive, and synergistic interactions.

$$\frac{1}{m_{add.}} = \sum_{i=1}^{n} \frac{1}{m_i} - \frac{n-1}{m_{neg.}} \tag{13}$$

$$\frac{m_{syn.}}{m_{neg.}} = \prod_{i=1}^{n} \frac{m_i}{m_{neg.}} \tag{14}$$

$$\lim_{n\to 0} \left(\frac{1 - m_{ant.}}{m_{ant.}} \right)^{1/n} = \lim_{n\to 0} \sum_{i=1}^{k} \left(\frac{1 - m_i}{m_i} \right)^{1/n} \tag{15}$$

Equation 15 should be equivalent to taking the maximum miRNA activity (minimal mKate2 expression) for constituent miRNAs at each level of EBFP2 expression, such that:

$$\frac{m_{ant.}}{m_{neg.}} = min \left\{ \frac{m_1}{m_{neg.}}, \ldots, \frac{m_k}{m_{neg.}} \right\} \tag{16}$$

Supplementary Note 2

One design consideration to note is that miRNA sensors bearing target sites in the 5' UTR can present challenges for a subset of miRNA target sites bearing an 'ATG' sequence (e.g. 176 of our 620 high confidence miRNA target sites contain an 'ATG'). In these cases, introduction of upstream open reading frames (uORFs) could result in reporter mKate2 knockdown that is not due to true miRNA activity. We tested several variants of sensors bearing miR-21-5p target sites (which do not contain a natural 'ATG' sequence) in the 5' UTR, and also added either no uORFs, out of frame uORFs, or in frame uORFs in order to determine the possible contributions to knockdown (Supplementary Fig. 16). By introducing additional nucleotides between miRNA target sites to place the uORFs in frame with mKate2 we observed that about half of the lost fluorescence could be recovered. In certain applications, this recovery may allow for miRNA targets bearing ATGs to be placed within the 5' UTR with small alterations.

Supplementary References

[1] Jonas, S. & Izaurralde, E. Towards a Molecular Understanding of microRNA-mediated Gene Silencing *Nat. Rev. Genet.* **16**, 421–433 (2015).

[2] Golden, R. J. *et al.* An Argonaute Phosphorylation Cycle Promotes microRNA-mediated Silencing. *Nature* **542**, 197–202 (2009).

[3] Rouya, c. *et al.* Human DDX6 Effects miRNA-mediated Gene Silencing via Direct Binding to CNOT1. *RNA* **20**, 1398–1409 (2014).

[4] Aeschimann, F. *et al.* LIN41 Post-transcriptionally Silences mRNAs by Two Distinct and Position-Dependent Mechanisms. *Mol. Cell* **65**, 476–489 (2017).

[5] Siciliano, V. *et al.* miRNAs Confer Phenotypic Robustness to Gene Networks by Suppressing Biological Noise. *Nat. Commun.* **4**, 2364 (2013).

[6] Wang, W. C. *et al.* miRExpress: Analyzing high-throughput sequencing data for profiling microRNA expression. *BMC Bioinform.* **10**, 328 (2009).

[7] Kozomara, A. & Griffiths-Jones, S. MiRBase: Annotating high confidence microRNAs using deep sequencing data. *Nucleic Acids Res.* **34**, 318–324 (2015).

[8] Darzacq, X. *et al.* In vivo dynamics of RNA polymerase II transcription. *Nat. Struct. Mol. Biol.* **14**, 796–806 (2007).

[9] Schwanhausser, B. *et al.* Global quantification of mammalian gene expression control. *Nature* **473**, 337–342 (2011).

[10] Raj, A., Peskin, C. S., Tranchina, D., Vargas, D. Y. & Tyagi, S. Stochastic mRNA synthesis in mammalian cells. *PLoS Biol.* **4**, 1707–1719 (2006).

[11] Dar, R. D. *et al.* Transcriptional burst frequency and burst size are equally modulated across the human genome. *Proc. Natl Acad. Sci. USA* **109**, 17454–9 (2012).

[12] Halter, M., Tona, A., Bhadriraju, K., Plant, A. L. & Elliott, J. T. Automated live cell imaging of green fluorescent protein degradation in individual fibroblasts. *Cytometry Part A* **71**, 827–834 (2007).

[13] Haley, B. & Zamore, P. D. Kinetic analysis of the RNAi enzyme complex. *Nat. Struct. and Mol. Biol.* **11**, 599–606 (2004).

[14] Bosson, A. D., Zamudio, J. R. & Sharp, P. A. Endogenous miRNA and target concentrations determine susceptibility to potential ceRNA competition. *Mol. Cell* **56**, 347–359 (2014).

[15] Chou, T. C. & Talalay, P. Generalized equations for the analysis of inhibitions of Michaelis-Menten and higher-order kinetic systems with two or more mutually exclusive and nonexclusive inhibitors. *Eur. J. Biochem.* **252**, 6438–6442 (1981).
