# Supplementary material for: A mixed antagonistic/synergistic miRNA repression model enables accurate predictions of multi-input miRNA sensor activity
Source: Nat Commun. 2018 Jun 22;9:2430. doi: 10.1038/s41467-018-04575-0 (PMC6014984; doi:10.1038/s41467-018-04575-0)
Supplement: Supplementary file 3 — Description of Additional Supplementary files [file 41467_2018_4575_MOESM3_ESM.pdf]

Description of Additional Supplementary Files

File Name: Supplementary Dataset 1
Description: Information concerning mature miRNA names (column A), miRNA target site sequence for a single repeat (column B), a barcode sequence included before each set of four target sites (column C), the forward sequence of the ultramer used for cloning steps (column D), and the reverse ultramer sequence (column E).

File Name: Supplementary Dataset 2
Description: Sequences and annotations in Genbank format for plasmids used in this study.

File Name: Supplementary Dataset 3
Description: Sequences for oligonucleotides used in this study. Oligonucleotide names (column A), sequences (column B), and information on their usage (column C) are provided.

File Name: Supplementary Dataset 4
Description: Matlab scripts used to analyze, model and predict flow cytometry data from miRNA sensors.
